# A Portable Electrospinner for Nanofiber Synthesis and Its Application for Cosmetic Treatment of Alopecia

**DOI:** 10.3390/nano9091317

**Published:** 2019-09-14

**Authors:** Richard A. Revia, Brandon A. Wagner, Miqin Zhang

**Affiliations:** 1 Department of Materials Science and Engineering, University of Washington, Seattle, WA 98195, USA; 2 Department of Neurological Surgery, University of Washington, Seattle, WA 98195, USA

**Keywords:** electrospinning, nanofibers, portable device, alopecia, cosmetics

## Abstract

A portable, handheld electrospinning apparatus is designed and constructed using off-the-shelf components and 3D-printed parts. The portable electrospinner is used to generate nanofibers with diameters ranging from 85 to 600 nm; examination of these fibers is achieved with scanning electron microscopy. This portable electrospinner has similar capabilities to standard stationary benchtop electrospinners in terms of the diversity of polymers the device is able to spin into nanofibers and their resulting size and morphology. However, it provides much more ambulatory flexibility, employs current-limiting measures that allow for safer operation and is cost effective. As a demonstration of the device’s unique application space afforded by its portability, the device is applied in direct-to-skin electrospinning to improve the aesthetics of simulated hair loss in a mouse model by electrospinning dyed polyacrylonitrile nanofibers that mimic hair. The superficial nanofiber treatment for thinning hair is able to achieve an improvement in appearance similar to that of a commercially available powder product but outperforms the powder in the nanofiber’s superior adherence to the affected area. The portable electrospinning apparatus overcomes many limitations of immobile benchtop electrospinners and holds promise for applications in consumer end-use scenarios such as the treatment of alopecia via cosmetic hair thickening.

## 1. Introduction

Electrospinning is a cost-effective technique for synthesizing polymeric fibers with diameters ranging from a few nanometers to tens of microns. Nanofibers electrospun with conventional benchtop electrospinners have found application in textiles [1,2], energy harvesting and storage [3,4,5,6], tissue engineering [7,8,9,10], packaging of pharmaceuticals [11] and wound healing [12,13,14,15] among others [16,17,18,19,20]. Although these traditional electrospinners can produce copious quantities of nanofibers, their large size and stationary design limit their utility in many key applications such as point-of-need nanofiber deposition (e.g., direct application of nanofibers to wound dressings in the clinic) and consumer end-user scenarios. These challenges may be readily addressed with a portable electrospinning device as described herein.

The popularity of electrospinning is largely due to the simplicity of the equipment. Conventional electrospinners primarily consist of three components [21]: a high voltage (HV) direct current (DC) source capable of supplying a voltage greater than 1 kV, a fluidic dispenser with a conductive tip (ordinarily a syringe and steel needle) and a collector (typically a grounded metal sheet such as aluminum foil). Here, we report on the development of a handheld, portable electrospinning device as an easy-to-use alternative to bulky, immobile benchtop electrospinners and demonstrate the functionality of this mobile device using the cosmetic treatment of alopecia as an example. The chief components of this design include both positive and negative HV DC-DC converters, a syringe pump driven by a stepper motor and a plastic enclosure. To minimize the cost of this portable electrospinner, the electrical system of the device is entirely constructed with off-the-shelf components, and structural elements are fabricated using a consumer-grade three-dimensional (3D) printer. Key electrospinning parameters (i.e., the syringe pump speed and the magnitude of the HV source) can easily be adjusted via a user-friendly interface integrated with a microcontroller (MCU). Most benchtop electrospinners use HV sources that are typically mains-powered devices and pose safety concerns due to their ability to draw large currents (>50 mA). The portable electrospinner device introduced herein avoids such hazards by isolating the HV circuit from all mains-powered devices and limits the HV sources from drawing no more than 2 mA of current.

An application of nanofibers electrospun by a portable electrospinning apparatus yet to be explored is the cosmetic treatment of alopecia by depositing dyed nanofibers onto the skin to simulate native human hair. Hair thinning, or male pattern baldness, affects half of all men over the age of 50 [22,23] and female pattern alopecia affects nearly a third of all women over the age of 30 [24]. Current therapies for alopecia include surgical transplantation of donor hair follicles to the scalp, medication with minoxidil or finasteride, or the use of cosmetic concealers such as a toupee or colored powder [23]. Each of these treatment options has its attendant limitations: the high cost and shortage of donor follicles hinder the surgical treatment recourse, the two U.S. Food and Drug Administration-approved drugs for alopecia do not always work or may not work to an acceptable degree and current cosmetic solutions lack aesthetic appeal. Here, we show how our device may be used to directly apply dyed nanofibers to the area of interest to overcome the above-mentioned limitations, which may open a new avenue for nanofiber application in the cosmetic treatment of alopecia.

## 2. Materials and Methods

### 2.1. Electrospinner Components and Fabrication

Two HV DC-DC converters, one supplying a positive voltage (Q101-5, XP Power, Singapore) with respect to ground and another supplying a negative voltage (Q101N-5, XP Power, Singapore), amplified a low voltage DC signal between 1 and 10 V to output an HV DC signal between 1 and 10 kV. Low voltage DC signals were generated using an ATmega2560 MCU (Microchip Technologies, Chandler, AZ, USA) in series with a non-inverting operational amplifier circuit with a gain of 2 V/V (OPA376AIDCK, Texas Instruments, Dallas, TX, USA) and an optocoupler (TIL111M, ON Semiconductor, Phoenix, AZ, USA). A custom-made syringe pump was used to push a polymer solution loaded in a syringe outfitted with a stainless-steel needle tip toward the HV source. The syringe pump was comprised of a 5 V DC power supply, 1 A stepper motor (QSH4218-35-10-027, Trinamic Motion Control, Hamburg, Germany), the Allegro A4988 motor driver (Allegro MircoSystems, Worcester, MA, USA) and structural components fabricated with a 3D printer (MakerGear M2, Beachwood, OH, USA) using 1.75 mm PLA filament. The enclosure for the handheld electrospinning apparatus was fabricated as well using the 3D printer and PLA filament.

A user-interface was constructed using PCB technology, a 24 × 2-character liquid crystal display (NHD-0224WH-ATDI-JT#, Newhaven Display International, Elgin, IL, USA) and a rotary encoder (PEC11R-4215F-N0024, Bourns Inc., Riverside, CA, USA). The device was either powered by 110 V AC mains electricity via a dedicated 24 V, 2.5A AC/DC adapter (BSG-60W2402500, Bosheng Electronic Technology Co. Ltd., Ningbo, China) or by four 9 V batteries (EN22, Energizer Battery Company, St. Louis, MO, USA). Power from either the 24 V adapter or the batteries was used to drive two voltage regulators: a 12 V regulator (μA7812, Texas Instruments, Dallas, TX, USA) and a 5 V regulator (MIC39100-5.0WS, Microchip Technology, Chandler, AZ, USA). In line with the power sources, and prior to the PCB, a 2-mA fuse was used for overcurrent protection (0273.002V, Littelfuse, Chicago, IL, USA).

### 2.2. Polymer Solutions

All chemicals were purchased from Sigma-Aldrich (St. Louis, MO, USA) unless otherwise noted. Polymer solutions for electrospinning were prepared by mixing the following polymers with their respective solvents at room temperature under constant stirring for 1 h prior to electrospinning: CA (average *M_n_* 30 kDa) dissolved in an acetone/deionized water mixture (4:1 *v*/*v*) at a concentration of 4% (*w*/*w*), PVA (average *M_w_* 93 kDa) dissolved in THF at 6% (*w*/*w*), PAN (average *M_w_* 150 kDa, Polysciences Inc., Warrington, PA) dissolved in DMF at 10% (*w*/*w*), PEO (average *M_v_* 900 kDa) dissolved in deionized water at 6% (w/w), PVDF (28-34 kP, Polysciences Inc., Warrington, PA) dissolved in a mixture of DMF and acetone (1:1 *v*/*v*) at a concentration of 15% (*w*/*w*) and PVP (average *M_w_* 1.3 MDa) dissolved in ethanol at 5% (*w*/*w*).

Two other polymer solutions were prepared as follows: PCL (average *M_n_* 80 kDa) was dissolved in TFE over a 24 h period under constant stirring at room temperature at a concentration of 15% (w/w); PS (average *M_w_* 350 kDa) was dissolved in THF over a 12 h period under constant stirring at room temperature at a concentration of 12% (*w*/*w*).

### 2.3. Electrospinning and Electrospraying

For all electrospinning and electrospraying experiments, a polymer solution was loaded into a 3 mL syringe (309657, BD, Franklin Lakes, NJ, USA) outfitted with a 20-gauge, stainless-steel needle with blunt tip (901-20-050, CML Supply, Lexington, KY, USA). A stainless-steel alligator clip (BU-60X, Mueller Electric Co., Akron, OH, USA) was used to connect the needle tip to the HV DC supply. The flow rate of the stepper motor powered syringe pump was set to 1 mL hr^−1^, and after a brief 1 second priming period, the HV source was adjusted until continuous nanofiber formation was observed.

Nanofibers and nanobeads were collected onto a grounded aluminum foil substrate placed 15 cm away from the needle tip. The connection between the collector and the ground plane of the electrospinning apparatus was maintained by an alligator clip and a cable soldered to a ground connection on the main PCB. For the experiment where nanofibers were electrospun onto skin, no ground cable was necessary to achieve nanofiber deposition directly onto the point of interest.

### 2.4. Scanning Electron Microscopy

Nanofibers collected on aluminum foil were mounted onto aluminum pin stubs (16111, Ted Pella Inc., Redding, CA, USA) and sputter-coated with Au/Pd for 50 s at 18 mA. SEM was performed using a FEI Sirion XL830 (FEI Company, Hillsboro, OR, USA) microscope at 5 kV accelerating voltage, a spot size of 3, and a 5 mm working distance. Nanofiber diameters were measured using ImageJ [25] by analyzing the diameters of 10 fibers from a representative 512 × 512 SEM image and computing the resulting average and standard deviation.

### 2.5. Determination of Solution Viscosity

The rheological properties of the polymer solutions were measured with a stress-controlled rheometer (MCR 301, Anton Paar, Graz, Austria). A parallel-plate geometry was used with a plate diameter of 25 mm. Viscosity was measured at a constant shear rate of 10 s^−1^ with a zero gap of 1 mm at 25 °C. Viscosity values are reported as the average and standard deviation of 10 measurements.

### 2.6. Hair Thickening

The solution for the electrospinning of dyed PAN nanofibers was prepared by dissolving 0.2 g of PAN in 1.8 g of DMF under gentle stirring for 5 min; to this solution, 0.1 g of Procion MX Dye 150 Jet Black (Jacquard Products, Healdsburg, CA, USA) was added under gentle stirring for 1 h. Hair thickening treatments were performed on deceased mice that were euthanized after their utilization in experiments approved by and in compliance with the University of Washington’s Institutional Animal Care and Use Committee’s guidelines. Square patches of fur, approximately 1 cm × 1 cm, were clipped from two mice. The commercial cosmetic powder Toppik™ was applied to the shaved area of one mouse. Dyed PAN nanofibers were directly electrospun onto the shaved area of the second mouse.

## 3. Results and Discussion

### 3.1. Electrospinner Design

**Figure 1:**
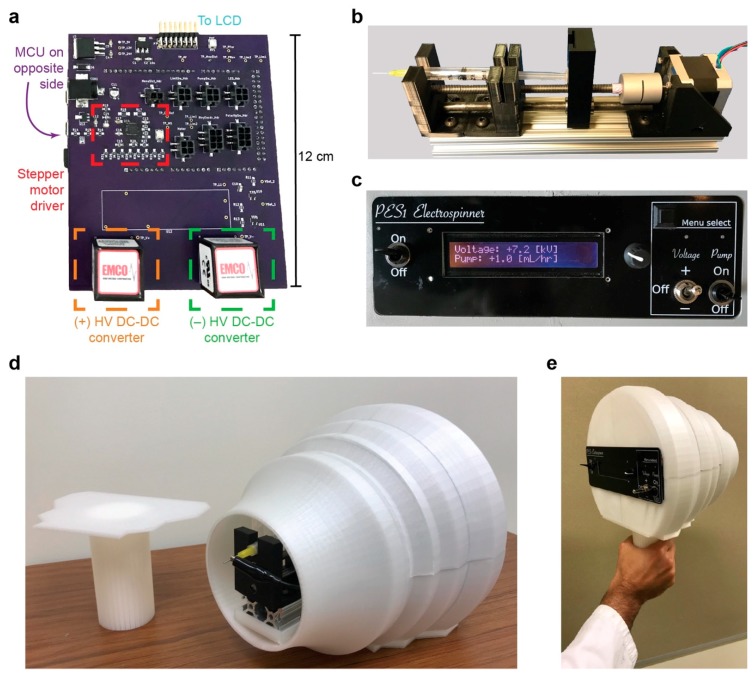
Handheld electrospinner device. (**a**) Main PCB with two HV DC-DC converters: positive (left) and negative (right). (**b**) Syringe pump driven by a stepper motor. (**c**) Front panel user interface. Fully constructed handheld electrospinning apparatus with (**d**) the handle detached and (**e**) the handle attached.

A typical benchtop electrospinning system comprises three key components: an HV electric potential source, a fluid feed system, and a fiber collector. In order to fabricate a miniaturized and portable version of the benchtop electrospinner, while keeping cost low and functionality high, easy to obtain commercially available electronic components were sought in our design and 3D printed parts were used to create structural elements. The main printed circuit board (PCB) is shown in Figure 1a, with major components and connections noted (a circuit diagram is provided in Figure S1 and a PCB schematic is provided in Figure S2). The MCU, not shown, is located on the back side of the PCB; the firmware loaded onto the MCU allows the user to control the flow rate of the syringe pump (Figure 1b) as well as the magnitude and polarity of the HV sources by interfacing with a rotary encoder and switches contained within the front panel (Figure 1c) of the device (a circuit diagram of the front panel is provided in Figure S3 and a PCB schematic is provided in Figure S4). The syringe pump is driven by a stepper motor and is housed in an enclosure made of 3D-printed poly(lactic acid) (PLA) parts. Figure 1d,e shows photographs of the handheld electrospinning device with the handle detached and with the handle attached, respectively. The electrospinner weighs 1.5 kg and has dimensions of 250 mm × 265 mm × 225 mm (or 350 mm when the handle is attached).

The apparatus can be operated at a voltage magnitude between 1 and 10 kV of either positive or negative polarity. A dual polarity voltage design was preferable due to evidence in the literature suggesting differences in the ease of synthesis [26], resulting morphology [27,28] and physical properties [29] of nanofibers electrospun with either positive or negative voltages. Some polymer solutions form superior nanofibers using a positive source voltage, whereas others are more easily generated using a negative voltage. The flow rate of the polymer solution may be adjusted between 0.5 and 10 mL hr^−1^ via the front panel. Both the HV sources and syringe pump are powered by low-voltage batteries. Syringes filled with polymer solution are easily loaded into the electrospinner by an access hatch at the top of the device. A ground connection port is provided on the front side of the device so a collector substrate may be held at 0 V potential relative to the HV source.

Due to the high voltages required for electrospinning, there are inherent safety concerns for users of any electrospinning apparatus. The matter of safety becomes especially important when designing electrospinners for use by untrained consumers. While the current rating of an electrical circuit is arguably the most salient metric of danger or safety, high-voltage circuits pose the risk of causing electric shocks regardless of the circuit’s current capacity. With these two aspects in mind—current and voltage—extra precautions were taken to limit the current drawn by the portable electrospinner. The portable electrospinner described draws a maximum 2 mA of current, and overcurrent protection is provided by a 2-mA fuse. When DC voltages in excess of 0.5 kV are involved, currents of 40 mA or less pose no serious hazard; conversely, currents of greater than 40 mA can cause involuntary muscle contractions, and currents of greater than 200 mA can induce fatal ventricular fibrillation [30]. With a 2-mA current limit in place, the portable electrospinner avoids any serious electrical hazard. However, because voltages required for electrospinning exceed 1 kV, the possibility of receiving a shock without even touching a conductive point on the circuit exists because the dielectric breakdown of air can occur in electric fields of magnitude near 3 kV mm^−1^. Thus, while the portable electrospinner cannot guard against all possibilities of receiving a shock, the current limiting measures taken ensure that the delivered current will be of such a small amount that no serious injury is likely.

### 3.2. Nanofiber Synthesis and Characterization

**Figure 2:**
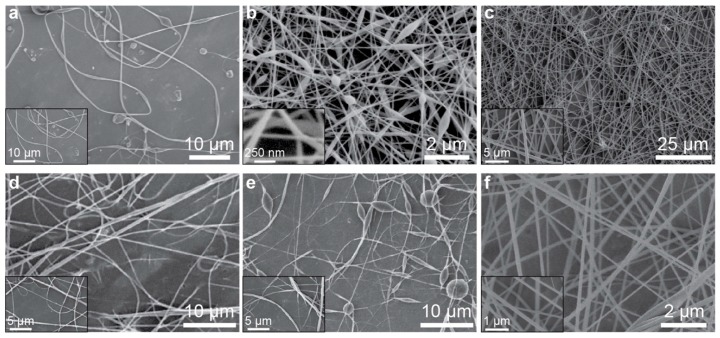
An array of nanofibers synthesized with the portable electrospinner: (**a**) 4 wt.% CA in acetone/water, (**b**) 6 wt.% PVA in THF, (**c**) 10 wt.% PAN in DMF, (**d**) 6 wt.% PEO in water, (**e**) 15 wt.% PCL in TFE and (**f**) 15 wt.% PVDF in DMF/acetone. Insets show SEM images of higher magnification.

**Figure 3:**
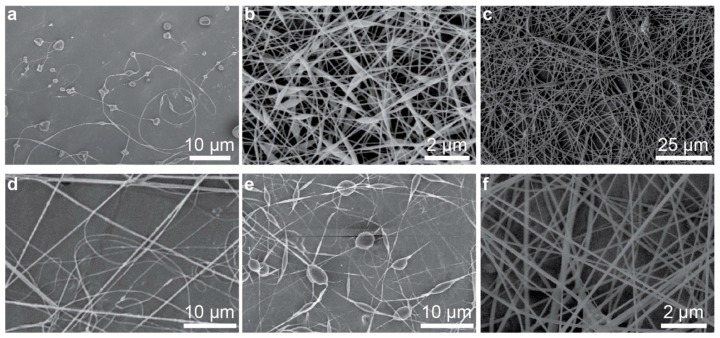
Array of nanofibers synthesized with a conventional benchtop electrospinner: (**a**) 4 wt.% CA in acetone/water, (**b**) 6 wt.% PVA in THF, (**c**) 10 wt.% PAN in DMF, (**d**) 6 wt.% PEO in water, (**e**) 15 wt.% PCL in TFE and (**f**) 15 wt.% PVDF in DMF/acetone.

To demonstrate the efficacy of the portable electrospinning apparatus, six polymer solutions were spun into nanofibers using the device. Polymers were dissolved in suitably volatile solvents and loaded into 3 mL syringes. The polymers selected include: cellulose acetate (CA) dissolved in an acetone/water mixture, poly(vinyl alcohol) (PVA) dissolved in tetrahydrofuran (THF), polyacrylonitrile (PAN) dissolved in dimethylformamide (DMF), poly(ethylene oxide) (PEO) dissolved in water, polycaprolactone (PCL) dissolved in 2,2,2-trifluoroethanol (TFE) and poly(vinylidene fluoride) (PVDF) dissolved in a mixture of DMF and acetone. After a brief 1 s priming period—which allowed a small bead of polymer solution to form at the needle tip of the polymer-loaded syringe—an HV DC signal was applied at the needle tip to induce electrospinning and the syringe pump was set to a constant flow rate of 1 mL hr^−1^. The magnitude of the HV signal was adjusted via the rotary encoder on the electrospinner’s front panel until a uniform and continuous flow of nanofibers were observed to emit from the needle tip; nanofibers were collected onto a grounded sheet of aluminum foil placed perpendicular to and 15 cm away from the needle tip. Table 1 provides information about the solutions used—including the polymer type and concentration, associated solvent, the voltage used for electrospinning and the diameters of the resultant nanofibers as measured using scanning electron microscopy (SEM). Nanofiber diameters are reported as the average and standard deviation of 10 fibers. Figure 2 displays high resolution SEM images of each of the polymeric nanofibers listed in Table 1; the morphology of each of the nanofibers synthesized is similar to the morphology of nanofibers obtained using traditional benchtop electrospinners, as shown in Figure 3. These results demonstrated that the handheld electrospinner can be used to synthesize nanofibers from a suite of polymers and obtain a wide range of fiber diameters.

Since the viscosity of the polymer solution significantly impacts how well the solution may electrospin into nanofibers, rheological measurements of all the polymer solutions used in this study were measured at a shear rate of 10 s^−1^ (Table 1). These data show the wide-ranging viscosities that the portable electrospinner is able to accommodate.

**Figure 4:**
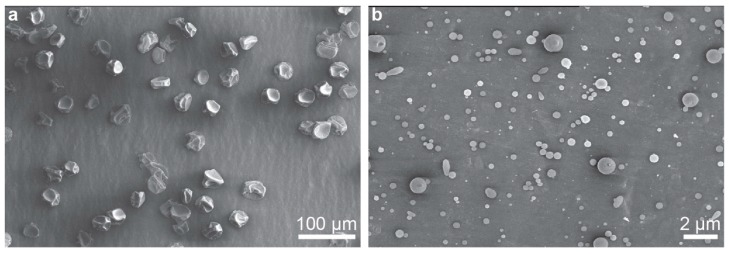
Beads electrosprayed from (**a**) 12 wt.% PS in THF and (**b**) 5 wt.% PVP in ethanol.

Not only can the device be used for synthesizing nanofibers, but it may produce nanobeads through electrospraying as well. Figure 4 depicts nanobeads produced from two polymer solutions: polystyrene (PS) dissolved in THF (Figure 4a) and polyvinylpyrrolidone (PVP) dissolved in ethanol (Figure 4b). The viscosities of the PS and PVP solutions were measured to be 4.6 × 10^−2^ ± 1.4 × 10^−3^ Pa⋅s and 3.2 × 10^−2^ ± 1.0 × 10^−3^ Pa⋅s, respectively.

### 3.3. Biological and Cosmetic Applications

Point-of-need electrospinning designates a situation where pre-fabricated nanofiber mats would prove insufficient or cumbersome because of the large size or irregular geometry of the area to be covered or due to the need to handle the nanofibers prior to application at the desired site. Conversely, a portable electrospinning apparatus that could be used to directly deposit nanofibers onto a large, arbitrarily shaped area overcomes such limitations.

**Figure 5:**
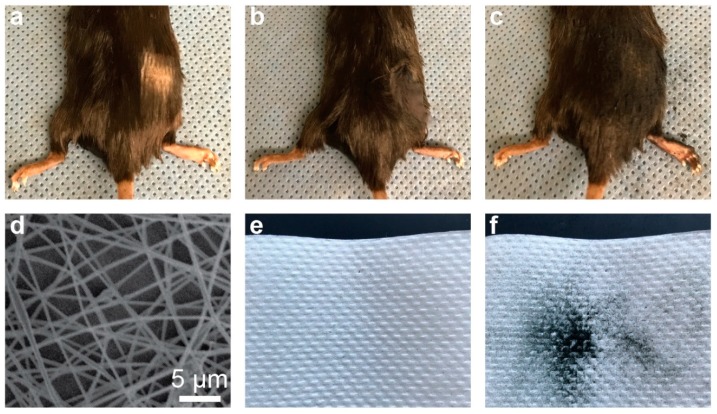
Application of the portable electrospinner for hair thickening. (**a**) A black-haired mouse with a small patch of hair removed. (**b**) Hair-thickening with black-dyed, electrospun PAN nanofibers. (**c**) Hair-thickening with a commercial powder product (Toppik™). (**d**) SEM image of the dyed PAN nanofibers. (**e**) Virtually no residue is left after contacting the nanofiber-treated skin to a napkin. (**f**) Significant residue left after contacting the Toppik™-treated skin to a napkin.

One example of point-of-need electrospinning is for the cosmetic treatment of alopecia. Nanofibers may prove to be an ideal cosmetic treatment for hair loss because the morphology of nanofibers may be tuned to be similar to that of human hair. Furthermore, nanofibers of an appropriate polymer may be dyed to match a wide variety of hair shades [31,32,33]. As a demonstration of the cosmetic benefit of using nanofibers synthesized with a portable electrospinner, PAN nanofibers were dyed black by mixing PAN polymer solution with a powdered textile dye prior to electrospinning. A small patch of fur was shaved off a mouse with black fur to simulate a person afflicted with alopecia (Figure 5a). Dyed nanofibers were then electrospun onto the area of skin devoid of hair. Figure 5b shows a photograph of the nanofiber treated area. For comparison, a commercial hair thickening powder—Toppik™—was applied to a second mouse that had a small patch of hair removed as well (Figure 5c). Comparing the photographs in Figure 5b,c, it is seen that nanofibers provide a similar aesthetic effect in concealing the bald region of skin as compared to the appearance of the powder-treated mouse. However, when the back of the mouse treated with nanofibers was placed against a paper towel and then removed, no residue was left behind (Figure 5e)—which means that nanofibers were firmly adhered to the bald skin. On the contrary, when we applied the same procedure to the mice treated with commercial powder, a large black stain was observed (Figure 5f). These results demonstrate the superiority of nanofibers over powder as a cosmetic treatment for alopecia as the nanofibers adhere better to the skin. An SEM image shows the morphology of dyed PAN nanofibers (Figure 5d). 

It should be noted that the results of this experiment are intended to serve as a proof-of-concept that nanofibers may be used to simulate human hair in the cosmetic application of nanofibers to hide an area of thinning hair. Before extending such a concept to use on humans, more study is required with respect to the biocompatibility and biodegradability of the nanofiber material employed. It is possible that an improperly engineered nanofiber material—when in contact with human skin—could produce an adverse reaction with the delicate biological environment presented by humans. 

## 4. Conclusions

We have constructed and demonstrated the use of a custom-designed portable electrospinning apparatus for direct-to-skin nanofiber generation for cosmetic hair-thickening treatments. The benefits of this electrospinning device over conventional equipment include: low cost, portable and handheld design, battery powered, composed of off-the-shelf components and dual polarity HV sources. A wide variety of polymeric nanofibers can be synthesized with the handheld electrospinner. The mobility of this handheld device was showcased in a point-of-need electrospinning scenario to generate dyed nanofibers for application in a cosmetic remedy for alopecia. The deposited nanofibers concealed an area of thinning hair and delivered aesthetically similar benefits as compared with a commercially available treatment option—Toppik™—while improving upon the adherence of the applied material to the site of interest. A portable electrospinning apparatus may prove to be highly appealing in direct-to-skin nanofiber deposition for both healthcare and consumer end-user scenarios.

## Figures and Tables

**Table 1:** Electrospinning parameters and measured nanofiber diameter.

Polymer	Solvent	Concentration	Voltage	Fiber Diameter	Viscosity
		(wt.%)	(kV)	(nm)	(Pa⋅s)
CA	Acetone/water (4:1 *v*/*v*)	4	9	600 ± 180	3.8 × 10^−1^ ± 1.4 × 10^−1^
PVA	THF	6	10	85 ± 20	2.3 × 10^−2^ ± 7.6 × 10^−2^
PAN	DMF	10	7	570 ± 75	3.2 × 10^−1^ ± 1 × 10^−3^
PEO	Water	6	8	570 ± 100	2.8 × 10^−2^ ± 8.4 × 10^−4^
PCL	TFE	15	5	320 ± 55	2.4 × 10^1^ ± 1.1
PVDF	DMF/acetone (1:1 *v*/*v*)	15	10	170 ± 45	5.8 × 10^−2^ ± 1.9 × 10^−3^

## Supplementary Materials

The following are available online at https://www.mdpi.com/2079-4991/9/9/1317/s1, Figure S1: Circuit diagram of the main PCB. Figure S2: Main PCB layout. Figure S3: Front panel circuit schematic. Figure S4: Front panel PCB design.
