# Supplementary material for: A Portable Electrospinner for Nanofiber Synthesis and Its Application for Cosmetic Treatment of Alopecia
Source: Nanomaterials (Basel). 2019 Sep 14;9(9):1317. doi: 10.3390/nano9091317 (PMC6781269; doi:10.3390/nano9091317)
Supplement: Supplementary file 1 [file nanomaterials-09-01317-s001.pdf]

nanomaterials

MDPI

Supporting Information

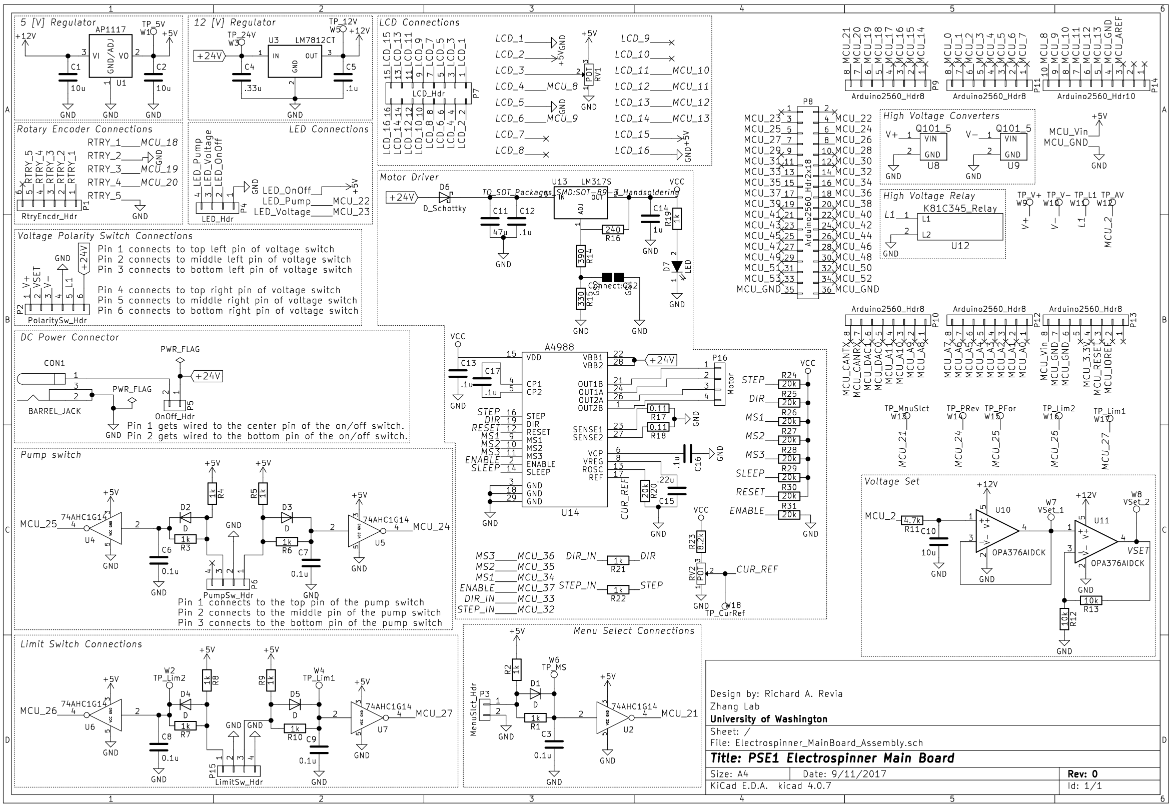

Figure S1. Circuit diagram of the main PCB.

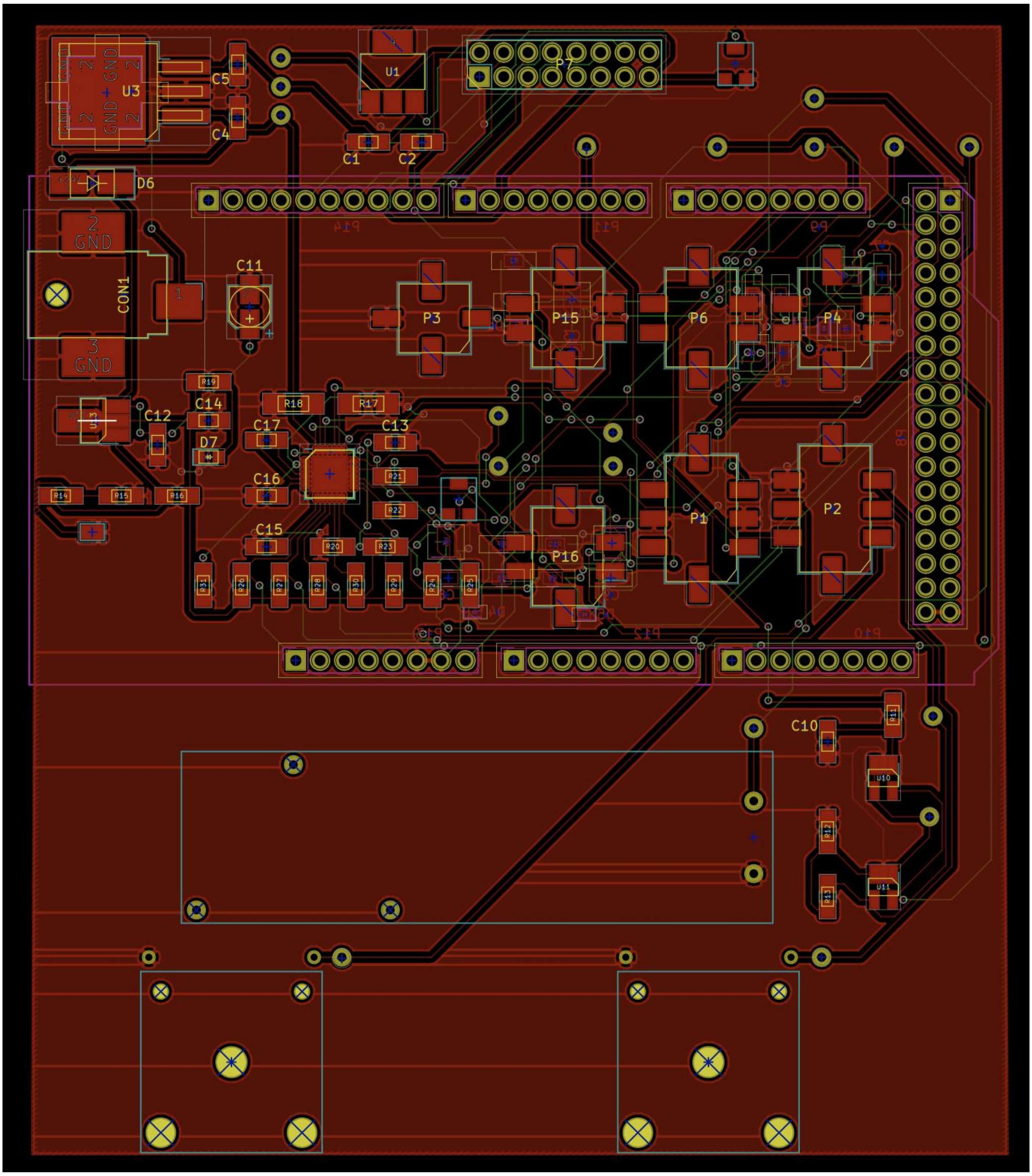

Figure S2. Main PCB layout.

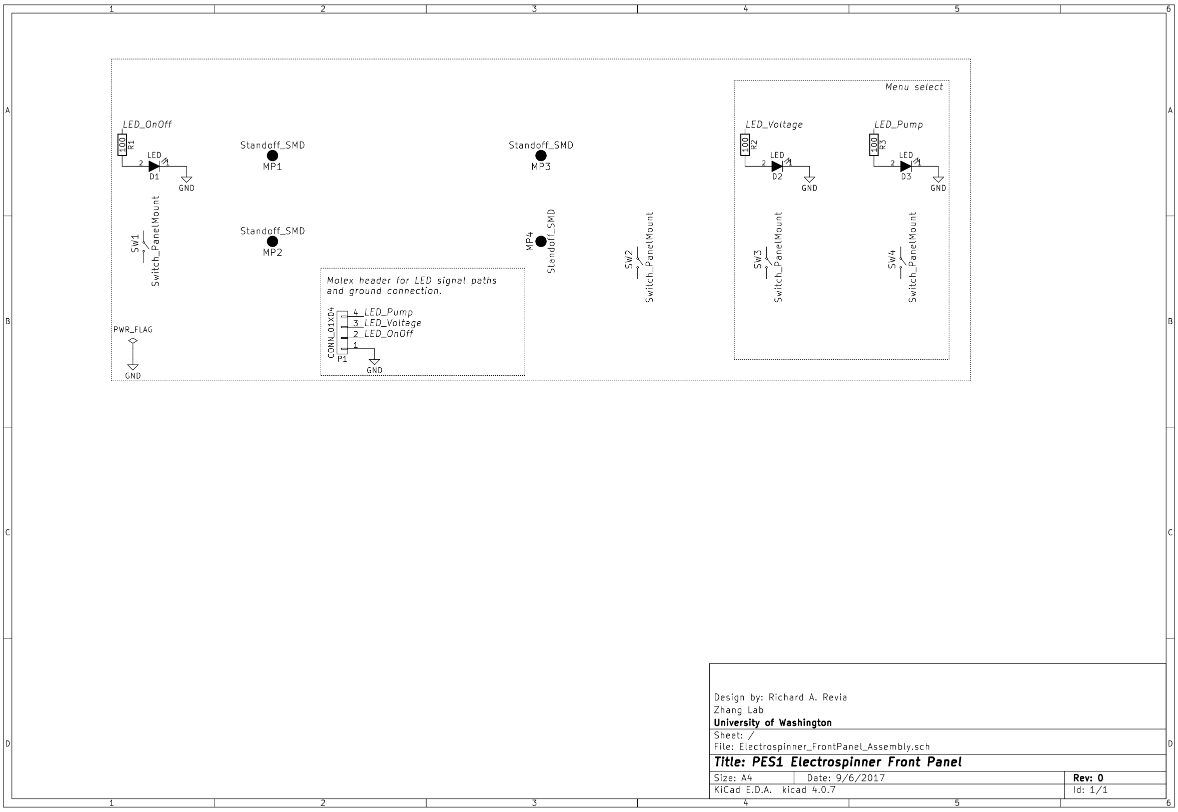

Figure S3. Front panel circuit schematic.

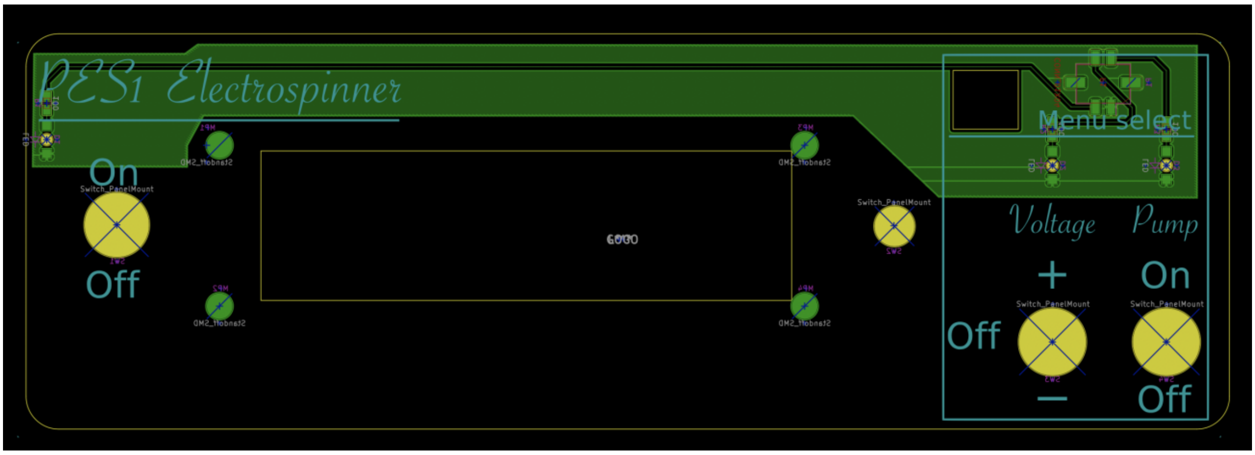

Figure S4. Front panel PCB design.
